# Visualization and Estimation of Nasal Spray Delivery to Olfactory Mucosa in an Image-Based Transparent Nasal Model

**DOI:** 10.3390/pharmaceutics15061657

**Published:** 2023-06-05

**Authors:** Amr Seifelnasr, Xiuhua April Si, Jinxiang Xi

**Affiliations:** 1 Department of Biomedical Engineering, University of Massachusetts, Lowell, MA 01854, USA; amr_seifelnasr@student.uml.edu; 2 Department of Mechanical Engineering, California Baptist University, Riverside, CA 92504, USA; asi@calbaptist.edu

**Keywords:** nasal spray, soft-mist inhaler, nose-to-brain (N2B), intranasal drug delivery, head position, vertex-to-floor, liquid film translocation

## Abstract

**Background:** Nose-to-brain (N2B) drug delivery offers unique advantages over intravenous methods; however, the delivery efficiency to the olfactory region using conventional nasal devices and protocols is low. This study proposes a new strategy to effectively deliver high doses to the olfactory region while minimizing dose variability and drug losses in other regions of the nasal cavity. **Materials and Methods:** The effects of delivery variables on the dosimetry of nasal sprays were systematically evaluated in a 3D-printed anatomical model that was generated from a magnetic resonance image of the nasal airway. The nasal model comprised four parts for regional dose quantification. A transparent nasal cast and fluorescent imaging were used for visualization, enabling detailed examination of the transient liquid film translocation, real-time feedback on input effect, and prompt adjustment to delivery variables, which included the head position, nozzle angle, applied dose, inhalation flow, and solution viscosity. **Results:** The results showed that the conventional vertex-to-floor head position was not optimal for olfactory delivery. Instead, a head position tilting 45–60° backward from the supine position gave a higher olfactory deposition and lower variability. A two-dose application (250 mg) was necessary to mobilize the liquid film that often accumulated in the front nose following the first dose administration. The presence of an inhalation flow reduced the olfactory deposition and redistributed the sprays to the middle meatus. The recommended olfactory delivery variables include a head position ranging 45–60°, a nozzle angle ranging 5–10°, two doses, and no inhalation flow. With these variables, an olfactory deposition fraction of 22.7 ± 3.7% was achieved in this study, with insignificant discrepancies in olfactory delivery between the right and left nasal passages. **Conclusions:** It is feasible to deliver clinically significant doses of nasal sprays to the olfactory region by leveraging an optimized combination of delivery variables.

## 1. Introduction

Nasal drug delivery has gained significant attention as a non-invasive method of delivering therapeutic agents to the brain. Drug administrations from the nose directly to the brain (known as N2B) have become an attractive substitute for conventional treatments of neurological disorders [1,2,3,4]. The olfactory mucosa, which is located in the upmost region of the nasal cavity, has been identified as a promising target for the administration of medications to the central nervous system (CNS). Neurological medications that are administered to the olfactory region have the ability to reach the CNS through the olfactory pathways and circumvent the Blood–Brain Barrier [5,6]. Nevertheless, the efficacy of N2B has been restricted due to the difficulties in administering clinically effective quantities of medication to the olfactory cleft using conventional nasal devices [7,8,9,10]. Thus, drug delivery to the brain through the nasal route is not commonly used in clinical settings due to the low amount of medication that can reach the olfactory region with conventional nasal delivery devices.

**Figure 1:**
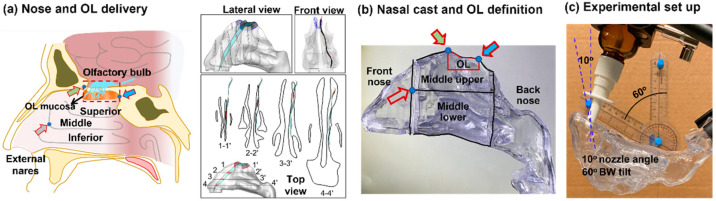
Nasal model and delivery experimental setup: (**a**) diagram of the human nose and olfactory (OL) region with anatomical features preventing effective olfactory delivery viewed from lateral, front, and top directions; (**b**) transparent nasal cast with four sections (front nose, middle upper (UpM), middle lower (LowM), back nose) and the delineated olfactory (OL) region using two landmarks (solid green and blue arrows); and (**c**) delivery experimental setup with adjustable nozzle angle (left panel) and head orientation (right panel).

The complex anatomy of the nasal cavity creates various obstacles that hinder the effective delivery of drugs through the olfactory route. As illustrated in Figure 1a, the inadequate delivery of drugs to the olfactory region results from the restricted airflow through this area, as well as the intricate structure of the nose, which screens out most intranasally delivered drugs well before they even get to the olfactory mucosa [11]. The nasal valve, the narrowest part of the nasal passage, acts as a filter that prevents most inhaled spray droplets from passing through. Those droplets that do make it past the valve tend to deposit within the labyrinthine turbinate area. As the airway transitions between the nasal valve and the turbinate region, the cross-sectional area increases, which reduces the respiratory flow velocity and facilitates the settling of large particles in the inferior and middle nasal cavity passages. The tortuous and restricted pathway of the turbinate area further promotes the deposition of administered droplets through direct interception and inertia-driven impaction. The superior meatus, situated at the uppermost part of the nasal cavity, has a narrower passage, making it difficult for respiratory air and particles to reach the olfactory mucosa [12]. Prior research has shown that the olfactory region receives less than 1% of aerosol medications that are administered intranasally [13]. González-Botas et al. found that when utilizing a typical radial-hole inhaler that dispenses 140 mL per application, most of the medication was deposited within the nasal valve and inferior meatus, with very little deposited in the olfactory cleft [14]. Accordingly, the significantly low amount of medication that can reach the brain through the nose has hindered the clinical use of N2B drug delivery and has become a significant obstacle in the advancement of neurological therapies via the nasal route [15].

The deposition of particles within the human nasal cavity has been extensively researched in human participants, as well as using in vitro nasal cavity models and computational simulations [16,17,18]. Although significant inter-subject variability exists in nasal morphology, research in this area has consistently demonstrated that several aspects—including the type of intranasal device used, medication formulation, delivery methods, as well as a patient’s inhalation pattern—can all have an impact on nasal deposition. Reliable techniques are available to determine the overall deposition fractions in hollow nasal replica casts, although substantiated techniques for visualizing and quantifying deposition fractions in specific regions or local areas are rare. However, when predicting clinical effects or assessing negative health outcomes, localized deposition is of greater clinical significance than total deposition. Computational models can predict deposition patterns, but the limited validation of these models and their inability to directly correlate with medical outcomes have hindered their use in clinical settings. Additionally, since multiple factors can impact spray deposition, and no single factor has been predominantly correlated with intranasal dosimetry, experimental testing is essential for validating targeted drug deliveries [6].

The bioequivalence of drugs administered within a specific region has been evaluated using techniques that make use of dyes such as methylene blue to inspect the intensity of staining [19]. However, these techniques have certain drawbacks, including the inability to quantify dosage accurately, as well as the diffusion and dripping of the solution. Some investigations employed gamma scintigraphy to visualize the distributions of deposition within human nasal cavities by using particles labeled with technetium-99m (99mTc) [20,21,22]. Gamma scintigraphy has also been utilized with in vitro nasal airway models [23,24,25]. In this method, a gamma camera is used to capture images of the deposition of radioactive aerosols, which are later analyzed and converted into dosage measurements based on color intensity. One major limitation of gamma scintigraphy is that its effectiveness is sensitive to the weakening of gamma rays as they pass through the body, resulting in up to 50% of photons either being dispersed or blocked by the surrounding tissue [26]. The scattering of photons can cause distortions in scintigraphy images and lead to inaccurate dose quantification, with around 30% of photons being scattered and causing measurement errors. Other factors that can affect accuracy include the dosage administered before the study, aerosol radiolabeling quality control, and radioactivity recovery [27]. Furthermore, scintigraphy images are only 2D and cannot distinguish between multiple layers of deposition. A cost-effective and efficient method was developed by Dalby and partners that utilized a water-detecting paste (Sar-Gel) to observe and measure the distribution of deposited water droplets. Kundoor and Dalby [28,29] have shown that Sar-Gel, which undergoes a color transformation from white to purple after coming into contact with water, is capable of detecting a volume of water as small as 0.5 μL, which corresponds to the minimum water droplet size found in nasal sprays. Sar-Gel was employed by Xi et al. to visualize and measure deposition in the nasal cavity and olfactory region in a sectional model of an adult nasal airway [6].

Different nasal devices and delivery methods have been studied to improve the delivery of therapeutic medication to the olfactory mucosa. Several important factors, such as exiting velocity, spray plume angle, droplet size, and angle of administration, affect the deposition location of nasal sprays [29,30,31,32]. Cheng et al. [30] examined the resulting deposition pattern from nasal spray pumps in a human nasal airway model and found that frontal nasal deposition was higher when larger spray particles and a wider spray angle were used, whereas smaller droplet sizes and a narrow spray cone angle resulted in a larger number of administered droplets passing through the nasal valve. Kundoor and Dalby [29] conducted a study to determine how nozzle orientation, ranging from 0 to 90°, affects olfactory delivery. They found that the most optimal deposition in the olfactory mucosa occurred when the nasal spray nozzle was oriented between 60° and 75°. Wang et al. [32] devised a method that involved intubation of the nozzle into the middle or superior meatus and administration of the spray directly below the olfactory mucosa. Although this approach had the potential to improve olfactory dosing, it involved invasive procedures that posed risks of tissue injury. As a result, this and similar techniques have not become popular. Gizurarson [31] proposed a less invasive technique that involved using an intranasal spray pump with a narrow cone angle to deliver medications to the olfactory region through the superior meatus. This method involved applying a relatively high-pressure spray to facilitate droplet penetration into the olfactory cleft. Nevertheless, the improvements in delivery to the olfactory region achieved through this approach were limited.

While there have been numerous test-based and computational studies into the deposition pattern of spray droplets, only a limited number of investigations explored the ensuing mobilization of these droplets once they have adhered to the walls of the nasal airway [12]. The migration of a liquid film due to gravity and/or flow shear following the application of a spray can result in a notable enhancement of delivering drugs to the olfactory mucosa. The effectiveness of topical treatments, such as intranasal drops, in reaching the olfactory cleft depends heavily on the position of the head during administration [33]. Merkus et al. [34] utilized methylene blue dye and nasal endoscopy to examine and compare four distinct head positions: upright, lying with the head tilted backward (Mygind), lying in a lateral position (Kaiteki), and head facing downward (vertex-to-floor, also known as Mecca). The researchers concluded that the vertex-to-floor orientation was the most effective in delivering spray doses to the upper nasal cavity and suggested it as the preferred one for intranasal spray administration targeting the olfactory cleft, particularly for individuals with nasal polyposis [34]. Likewise, when Cannady et al. [35] administered three dexamethasone drops intranasally to the vestibules of patients who had undergone endoscopic sinus surgery, they found that maintaining a vertex-to-floor head orientation resulted in effective delivery to the maxillary, ethmoid, and sphenoid sinuses, as well as the olfactory mucosa in a consistent manner. Furthermore, it was found that holding the vertex-to-floor head orientation for a 5 min duration resulted in higher levels of olfactory deposition compared to retaining the position for only 1 min, which suggests that the translocation of the liquid film can occur within the first 5 min following the application of the nasal drop [35]. In a study conducted by Milk et al. [33], they compared the delivery of intranasal drops to the olfactory region using two separate head positions: lying with the head tilted backward and vertex-to-floor. The study found that these two head positions resulted in similar levels of drug delivery to the olfactory mucosa [33]. Mori et al. [36] performed a study in which they evaluated the effectiveness of the Kaiteki orientation (which involves lying in a lateral position tilting the head and lifting the chin) for delivering intranasal sprays to the olfactory mucosa in healthy individuals. The results showed that the nasal spray was able to get to the olfactory cleft among 96% of decongested subjects and in 75% of those who did not receive treatment [36].

The above-mentioned studies utilized a variety of techniques to visualize the distribution and deposition patterns of sprays administered into the nasal cavity, which makes it challenging to compare them to one another [23]. Moreover, the evaluation of subregional doses in some of those studies was based on subjective evaluations by the researchers, which can make it difficult to compare results across different groups. This qualitative approach to assessing doses can create further confusion in interpreting the data [37]. Additionally, in order to establish a correlation between dose and therapeutic response for the treatment of olfactory impairment or neurological disorders, it is crucial to have accurate quantitative information about the dose delivered to the olfactory region. An alternative method to overcome these challenges involves utilizing transparent multipiece nasal cavity casts in studies pertaining to in vitro intranasal drug delivery, similar to the one used in this study. Such models provide clear and detailed views of the deposition patterns of administered nasal sprays. They allow for visualization of the distribution of nasal sprays throughout the different parts of the nasal cavity, as well as more accurate quantification of the deposition pattern of nasal sprays. Making use of such nasal cavity casts can allow researchers to assess the nasal spray distribution pattern throughout the nasal cavity with greater precision, better optimize nasal spray delivery, and evaluate the effectiveness of different drug formulations.

A good delivery protocol for olfactory (OL) delivery should (1) deliver clinically significant doses to the OL region, (2) have low variability in OL dosimetry, (3) minimize wastes in other regions, and (4) be easy to use. In our previous studies, it has been demonstrated that the dosimetry of nasal spray delivery was determined not only by the initial deposition of spray droplets but also by the subsequent liquid film translocation, which further depends on many factors such as gravity, fluid viscosity, wall angle, etc. [38]. With a transparent nasal cast and fluorescence, it now becomes feasible to visualize the dynamic process of the liquid formation and translocation under different delivery scenarios and thus identify the optimal delivery protocol to the target. Specific to the challenges that have precluded effective OL drug delivery, this visualization method allows a detailed examination of the spray liquid film migration toward the OL region in a fine spatiotemporal manner. It is thus promising to (1) evaluate the relative effects of individual delivery variables on the dynamic spray liquid film behavior and final OL dosimetry, and then (2) put together the acquired knowledge and recommend a new delivery method for improved OL delivery.

The objective of this study was to establish a technique using a soft-mist inhaler that can effectively administer medications to the olfactory mucosa for the treatment of neurological disorders and brain tumors. An anatomically accurate, transparent nasal cavity model was used to test the influence of a variety of delivery parameters, including the head position, nozzle angle, dose number, flow rate, and solution viscosity, on the spray deposition pattern and identify the optimum spray delivery method targeting the olfactory region. Specific aims include:

(1) Visualize the deposition distribution of intranasally administered sprays and subsequent liquid film translocation in the nasal cavity using different angles of administration, head positions, number of spray applications, and inhalation flow rates.
(2) Visualize the effect of formulation viscosity on the dosimetry of intranasal sprays.
(3) Quantify the deposition of intranasal sprays in the olfactory cleft, the turbinate region, the front nose, as well as the nasopharynx.
(4) Examine the results and compare the performance between the different test cases to determine the optimal combination of factors that lead to maximum bioavailability in the olfactory region (i.e., delivery of clinically significant doses).

## 2. Materials and Methods

### 2.1. Nasal Cast Model

The nasal airway model that had been reconstructed from MRI scans of a 53-year-old male was used for the in vitro deposition tests [39]. This nose model has been extensively used in previous studies for deposition experiments [39,40,41,42,43,44,45] and computational simulations [46,47,48,49]. Transparent hollow casts were fabricated with a Polyjet 3D printer (Stratasys, Inc., Rehovot, Israel) with a printing resolution of 30 µm and a Somos WaterShed XC 11122 stereolithography (SLA) material (Figure 1). To measure regional deposition, each cast was separated into four parts, i.e., the front nose (vestibule and valve), upper middle (superior meatus), middle lower (middle and inferior meatus), and back nose (caudal turbinate and nasopharynx). To secure a good seal, a stepped groove was created at all connecting ends in each part. The olfactory region was defined as the area that houses the olfactory nerves and enables nose-to-brain drug transport (Figure 1a). Two landmarks were used, with the nose apex defining the front and the second peak defining the back of the olfactory region (red line, Figure 1b). The area of the olfactory region was around 20% of the middle upper nose and 8.7% of the middle nose. 

### 2.2. Study Design

To study the effects of the head position on OL delivery, the vertex-to-floor was first tested with four nozzle angles (0°, 5°, 10°, 15°) counterclockwise from the nostril normal. This position has been commonly assumed to be optimal for OL delivery based on the intuition that nasal sprays would settle down to the OL region, which is presumably the lowest point in the nasal cavity, by gravity. The spray solution was a water solution with 0.4% *w*/*v* methyl cellulose (MC). Two doses would be applied in the nose with no inhalation flow. A video camera operating at 30 frames per second was used to document the motion and deposition of the spray liquid film in each test. Both the deposition fraction (DF) and variability in the OL region were quantified.

Three other head positions were then tested: 60°, 45°, and 30° backward (BW) tilt from the supine position. Note that the vertex-to-floor position was equivalent to tilting 90° BW from supine, even though it was more often practiced by taking a praying position. Four nozzle angles (0°, 5°, 10°, 15°) were tested with a 60° BW tilt head position in order to narrow down the optimal delivery variables for the head position and nozzle angle through the comparison of liquid film transient behavior, OL dose/variability, and drug losses in other regions. Two nozzle angles (5°, 10°) were tested for the head positions of 45° and 30° BW tilt. Considering that the slope of the nasal roof became increasingly steeper from the vertex-to-floor to 30° BW tilt, it was hypothesized that a steeper slope would increase the liquid film flowability and OL delivery, but it would also increase the risk of liquid overflow from the OL region and thus the drug loss in the nasopharynx. 

To study the inhalation flow effects on the spray liquid film behavior and fate, two flow rates, 10 and 20 L/min, were tested separately with the head tilted BW 45° and 60°. Considering that the increased flow shear could facilitate the liquid film motion, the OL DFs were quantified following a single-dose administration as well as a two-dose administration. This would help answer whether a combination of head position, nozzle angle, and flow shear is sufficient to effectively deliver nasal sprays to the OL region.

Considering that the solution viscosity could affect the liquid film translocation and thus OL delivery, four more spray solutions with different methyl cellulose (MC) concentrations (0.1%, 0.2%, 0.3%, and 0.5% *w*/*v*) were considered in addition to the baseline case (0.4% *w*/*v* MC). The solution viscosity of varying MC concentrations was measured using a digital rotational viscometer (Cgoldenwall, NDJ-5S, Hangzhou, China).

Considering that the liquid film translocation was sensitive to the nasal anatomy, differences in OL delivery between the right and left nasal passage were tested in the left nose with a head 60° position and two nozzle angles (5° and 10°). The nasal cast used in this study was created from MRI images of a 53-year-old male, and the two nasal passages had different morphologies [50]. The right–left discrepancy in transient liquid film behavior and eventual OL deposition would shed light on the OL DF variability due to geometry variations.

Considering the liquid evaporation effects, the following procedures were taken to estimate and minimize the evaporation effect on the olfactory dosimetry. Prior to the in vitro tests, the spray mass per application from the inhaler was quantified by weighing (1) the mass difference of the inhaler before and after the application, and (2) the mass of sprays collected in the in vitro cast model with no inhalation flow immediately after the application. In the second case, all administered sprays would be deposited in the cast model. We observed an insignificant difference (<1%) between these two cases. Considering that a longer time was needed for in-deposition tests with sectional cast models (i.e., model dissembling and part weighing), evaporation was more likely to happen. Immediately after each test, the model parts were promptly disassembled and weighed to minimize evaporation. In addition, evaporation occurred for all parts. Thus, its impact on the relative deposition (i.e., deposition fraction) presented hereof was expected to be insignificant. 

### 2.3. Inhaler and Spray Solutions

A refillable soft-mist spray inhaler (Hengni) was used to atomize a spray solution with 0.4% *w*/*v* methyl cellulose (MC) concentration. The spray discharge from the inhaler and subsequent spray plume development in the open space were captured at a rate of 2000 frames per second with a Phantom VEO 1310 camera (Vision Research Inc., Wayne, NJ, USA). 

### 2.4. Protocol for Nasal Spray Delivery

The experimental setup is shown in Figure 1c. Soldering clamps were used to fix the assembled nasal cast and soft mist inhaler to prescribed head positions and nozzle angles. The inhaler nozzle was inserted 1 cm into the right nostril. The nozzle angle was defined as counterclockwise from the nostril normal and was controlled using a protractor prior to spray administration, as illustrated in Figure 1c. Similarly, the head position was defined as a backward (BW) tilt from the supine position and was controlled using a protractor. When multiple doses were applied, a waiting period of 5 s was observed between the two spray applications. A vacuum (Robinair 3 CFM, Warren, MI, USA) was employed to generate the inhalation flow, and a flow meter (Omega, FL-510, Stamford, CT, USA) was utilized to regulate the flow rate. 

In order to examine the transient liquid film translocation within the nasal cavity, a solution containing 0.5% fluorescent dye (GLO Effex, Murrieta, CA, USA) was used for visualization, which exhibited a bright green color when exposed to a 385–395 nm LED light. The entire process, from nasal administration to one minute after spray administration, was captured with a video camera at 30 frames per second.

To quantify the deposited doses in different regions of the nose (i.e., front, middle upper, middle lower, and back), the masses before and after each run were measured with an electronic scale with a range/resolution of 120 g/0.0001 g (Bonvoisin, San Jose, CA, USA). The deposition fraction (DF) was calculated as the percentage of the regional dose over the applied dose. To calculate the mean and variability, each test was conducted a minimum of three times. 

### 2.5. Image-Based Estimation of Olfactory Dosimetry

There were challenges in quantifying the olfactory (OL) dose using the current mass-measuring method. When a continuous liquid film covered the olfactory region and its neighboring region, potential liquid film relocation during disassembly would make the dose measurement unreliable. As a result, an image-based method was applied in this study to estimate the OL dose. First, the projected area of the liquid-covered region (A1) in the middle upper nose was quantified on an image using a user-defined MATLAB code. The dose per unit liquid-covered area was calculated as the deposited dose in this region over A1. Similarly, the liquid-covered region (A2) in the OL region was also quantified. The OL dosimetry was then estimated as the product of A2 and dose per unit area in the liquid-covered region, i.e., A2 × (middle-upper-dose/A1). This method was based on an assumption of equal (or similar) dose per unit area in the liquid-covered region between the middle upper nose and olfactory region; this assumption appeared reasonable based on the deposition images, which are presented in later sections. 

### 2.6. Statistical Analysis

Minitab (State College, PA, USA) was employed to perform statistical analyses of the deposition results (outputs) and determine the importance of different delivery variables (inputs). The deposition fraction variability was assessed using one-way analysis of variance (ANOVA). A mean and standard deviation were calculated for the deposition fractions. 

## 3. Results

### 3.1. Characterization of Spray Viscosity and Aerosol Generation

**Figure 2:**
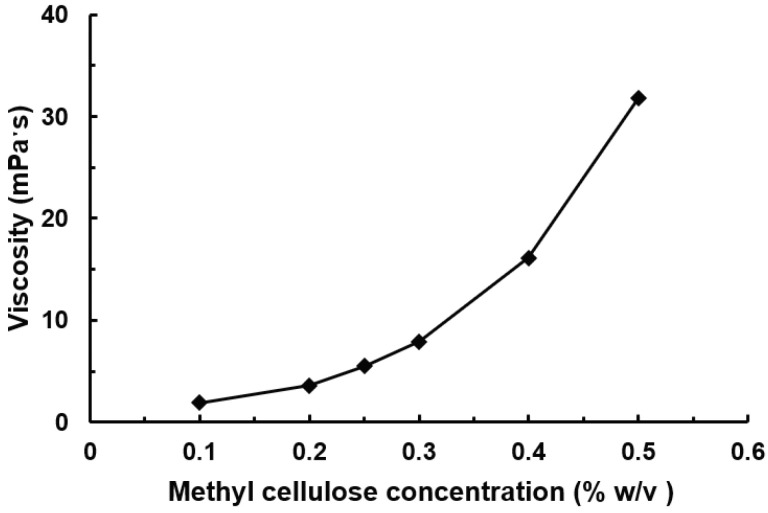
Measured viscosity of nasal spray solutions with varying methyl cellulose concentrations (% *w*/*v*).

Figure 2 presents the measured viscosity of nasal spray solutions with varying methyl cellulose (MC) concentrations (% *w*/*v*). The solution viscosity increased nonlinearly with increasing MC concentration. The viscosity was measured to be 1.9 mPa·s at an MC concentration of 0.1% *w*/*v*, 16.1 mPa·s at 0.4% *w*/*v*, and 31.8 mPaˑs at 0.5% *w*/*v*. 

**Figure 3:**
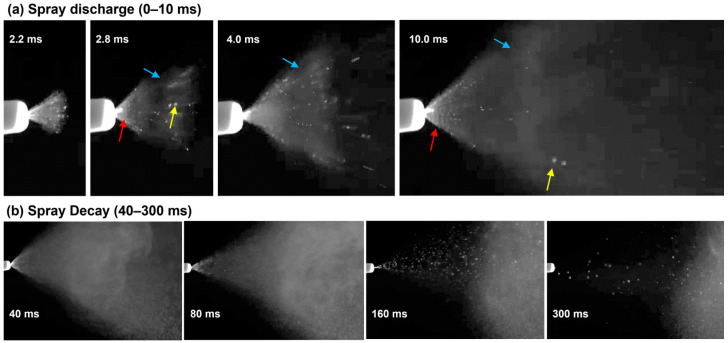
High-speed images of the spray droplets from the soft mist inhaler during the first 300 ms after actuation in two stages: (**a**) spray discharge (0–30 ms) and (**b**) spray plume decay (30–300 ms).

Figure 3 shows the high-speed images (recorded at 2000 fps) of the spray aerosols from the soft mist inhaler during the first 300 ms after actuation. Two stages were observed: the forceful discharge of spray droplets during 0–30 ms, and the spray plume decay during the remaining 30–300 ms. At the very beginning of the actuation (2.2–4 ms), polydisperse spray droplets were generated, with liquid filaments immediately outside of the nozzle orifice (red arrow) and occasional large droplets further downstream (yellow arrow). Due to the resistance of the ambient air, a vortex ring was observed to form at the front of the soft mist at 2.8 ms (blue arrow), which moved forward at a slower pace (blue arrows, 2.8–10 ms) in comparison to the large droplets or even the soft mist as a whole (Figure 3a).

At the spray decay stage (40–300 ms), the spray plume angle decreased gradually, and the fraction of large spray droplets increased (Figure 3b), indicating decreasing actuation energy. From 160 ms, the actuation energy was no longer sufficient to generate a soft mist, leaving only large droplets being released. At 300 ms, even the generation of large droplets stopped.

### 3.2. Vertex-To-Floor Head Position for Olfactory Delivery

**Figure 4:**
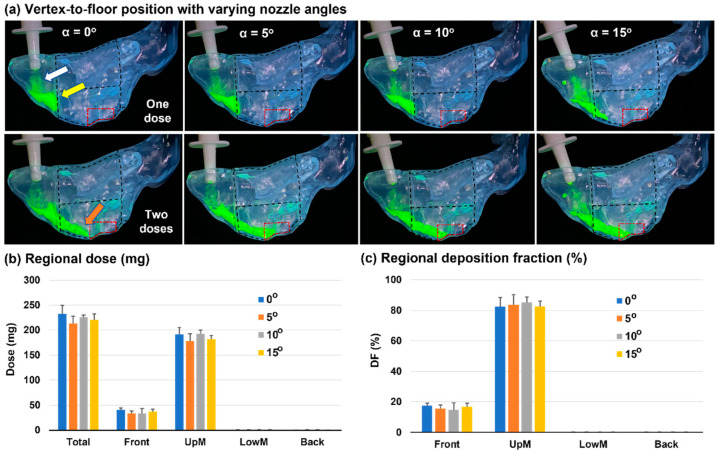
Nozzle angle effects on the nasal spray dosimetry in a nasal cast with a vertex-to-floor head position: (**a**) fluorescent visualization of the spray distributions after one and two actuations using different nozzle angles (α: 0–15°); (**b**) quantification of total and regional deposition after two doses; and (**c**) deposition fractions (DF) in four nasal regions using different nozzle angles (α: 0–15°). UpM: middle upper; LowM: middle lower.

Figure 4 shows the spray dosimetry in a nasal cast with a vertex-to-floor head position with four nozzle angles (i.e., 0°, 5°, 10°, 15° relative to the nostril’s normal direction). It was observed that one dose only was not enough to dispense drugs to the OL region, with nearly all sprays stuck in the top front nose (first row, Figure 4a). This might result from the narrow passages of this region, as well as the high liquid viscosity (cohesive inter-molecular force), wall adhesion (liquid–wall), and surface tension (liquid–air). The nasal vestibule and valve were very confined spaces. Due to the high inertia of discharged spray droplets, they were deposited on the lateral walls (white arrow) and roof of the top front nose (yellow arrow), particularly those of the nasal valve. The deposited droplets coalesced into a focused continuous liquid film, which, with collective gravity and interfacial forces, could behave differently from those of individual droplets. Once stability was reached, the gravity and inertia of the liquid film were insufficient to break this stability and mobilize the liquid film.

The second row of Figure 4a shows the spray distribution one minute after applying the second dose. The added mass destabilized and mobilized the liquid film. Once the film translocation started, its own inertia would keep it moving forward till the stabilizing forces slowed it down, and a new force equilibrium was reached. It was noted that a dynamic shear was often smaller than its static counterpart. As the liquid film expanded further, its thickness decreased, and the added mass effect dwindled until the liquid stopped moving (brown arrow). It was further noted that the gravity effect depended on the local incline angle. As illustrated in Figure 4a, at a vertex-to-floor position, the superior meatus acted as an inclined furrow, which became progressively flatter from the nasal valve to the nasal crest. After the crest, the furrow reverted to an upward angle so that gravity would slow down the film motion and stabilize it to the furrow valley (nasal crest herein). 

A quantitative comparison of total and regional deposition among different nozzle angles is shown in Figure 4b. Overall, similar doses were delivered to the front nose and the middle upper nose (UpM). Nearly no dose was deposited in the middle lower nose (LowM) and back nose. Figure 4c shows the normalized deposition by the total dose (i.e., deposition fraction, DF). In this case, about 82.6–85.2% of the applied dose reached the middle upper nose. Only a small fraction reached the olfactory region, which was quantified, as will be shown later, using an image-based method.

### 3.3. Head Position: 60° Backward Tilt from the Supine Position

#### 3.3.1. Deposition Distribution

**Figure 5:**
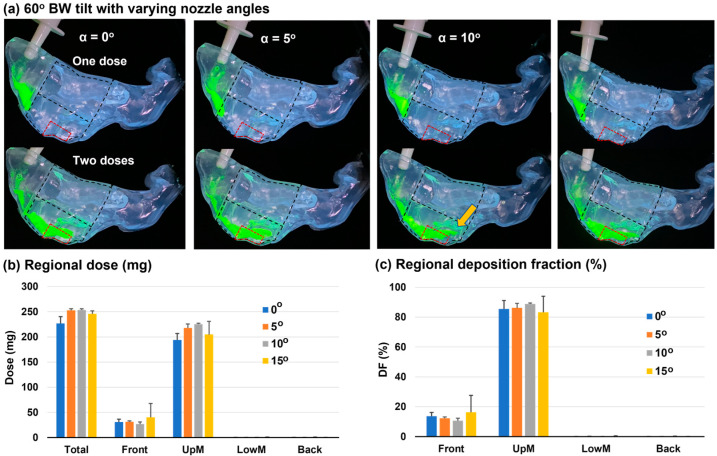
Nozzle angle effects on the nasal spray dosimetry in a nasal cast with a head position tilted backward 60° from the supine position (i.e., 60° BW tilt): (**a**) spray distribution after one and two actuations for nozzle angles α of 0–15°; (**b**) quantified depositions after applying two doses; and (**c**) deposition fractions (DF) in four nasal regions for nozzle angles α: 0–15°. UpM: middle upper; LowM: middle lower.

To improve OL delivery, the head position was adjusted to be 60° backward tilt from the supine position (referred to as 60° BW tilt hereafter). At this position, the roof of the front nose had a larger incline angle and, thus, a larger gravitational effect. As expected, the liquid film after the first dose moved slightly further downward than the corresponding vertex-to-floor position (Figure 5a vs. Figure 4a, first row). Furthermore, the liquid film after the second dose reached the posterior OL region (brown arrow) and, therefore, improved the delivery to the OL region more than the vertex-to-floor head position, given all other factors the same (Figure 5a vs. Figure 4a, second row). 

Similar to the vertex-to-floor position, nearly no deposition was observed in the middle lower nose or the back nose (Figure 5b,c). A slightly higher percentage of applied dose reached the middle upper nose (i.e., UpM DF: 83.3–88.8%) than the vertex-to-floor position (Figure 5c vs. Figure 4c). For the nozzle angle of 5–10°, the 60° BW tilt head position led to much lower variability in the UpM DF than the vertex-to-floor position, as evident by the lower standard deviation of 0.7–3.0% in Figure 5c vs. 3.5–6.7% in Figure 4c.

The liquid film coverage appeared to be sensitive to the nozzle angle when adopting a 60° BW tilt head position. From Figure 5a, there was more coverage of the olfactory region at the nozzle angle of 5–15° than at 0°. The nozzle angle affected the initial deposition position and perhaps the initial liquid film inertia as well. The spray plume traveled longer in the nose before deposition when discharged from a more inclined nozzle, which might increase the liquid film flowability and allow it to move further (Figure 5a).

#### 3.3.2. Dynamic Formation and Translocation of Liquid Film

**Figure 6:**
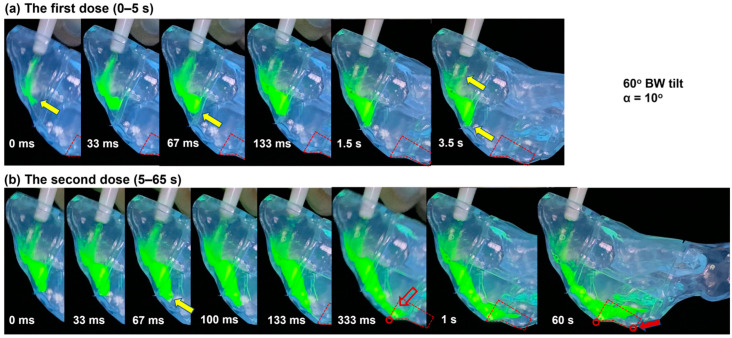
Snapshots of the dynamic liquid translocation in the nasal cast with a 60° BW tilt head position from supine and a nozzle angle of 10° from the nostril normal (**a**) after the first dose (0–4 s), and (**b**) after the second dose (0–60 s, or 4–64 s if counted from the first dose).

To better understand the dynamic process of the formation and translocation of liquid film, a time series of spray deposition snapshots are plotted in Figure 6a,b at varying instants after applying the first and second dose, respectively. The head position was 60° BW tilt, the nozzle angle was 10°, and the images were acquired at 30 fps. During the first 0–67 ms, the high-speed spray impinged on the two lateral walls and roof of the front nose (yellow arrows, Figure 6a). A liquid film formed on the inclined wall of the front nose roof upon contact (0 ms, Figure 6a). With continuous sprays from 33 ms to 67 ms, the liquid film grew in size and started to move downward along the inclined wall due to the self-weight and the impaction inertia. A large, rounded liquid front formed because of the liquid–wall adhesion and liquid–air surface tension. From 133 ms, when the spray discharge was completed, the liquid front continued to move along the inclined wall, but at a much slower speed. After the completion of the spray discharge at 100 ms, the liquid front continued to advance along the inclined wall, albeit at a significantly reduced pace (Figure 6a, 0.133–3.5 s). Only a short distance (less than 1 cm) was traveled within the next 4 to 5 s, reflecting an equilibrium state between the destabilizing gravitational force and the resistant forces such as wall adhesion, viscosity, and surface tension.

The dynamic progression of the liquid film after the second dose is displayed in Figure 6b within one minute. Around 125 mg of the spray formulation was added to the existing liquid film between 0 and 100 ms (yellow arrow). The added mass and kinetic energy mobilized the otherwise static liquid film, which was observed to move at an increasing speed from 100 to 333 ms, followed by a deceleration. At 333 ms after the second dose (hollow red arrow, Figure 6b), the liquid film reached the nasal crest, which was also one of the two landmarks defining the olfactory region. After 60 s, the liquid film crawled beyond the second landmark (solid red arrow, Figure 6b) and covered the entire OL region. 

### 3.4. Head Positions of 45° and 30° Backward Tilt from the Supine Position

**Figure 7:**
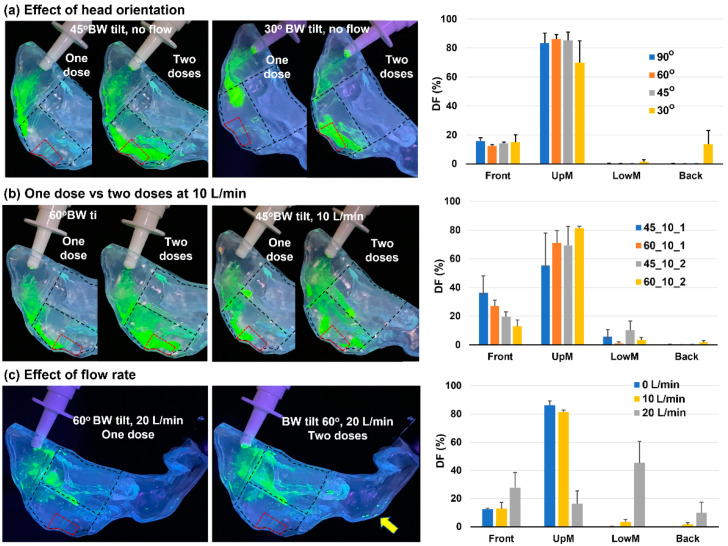
Effects of delivery variables (head orientation, dose number, and inhalation flow rate) on the regional spray dosimetry: (**a**) head orientation of 45° and 30° BW tilt from the supine position (no flow); (**b**) effect of 10 L/min inhalation flow rate and dose number; (**c**) spray dosimetry at 20 L/min. Here, 45_10_1: head orientation of 45°, inhalation rate of 10 L/min, and 1 dose; 45_10_2: head orientation of 45°, inhalation rate of 10 L/min, and 2 doses; similarly for 60_10_1 and 60_10_2.

Further tests were conducted with the head position being adjusted to 45° and 30° BW tilt from the supine position, as shown in Figure 7a. Both had a nozzle angle of 10° and an inhalation flow rate of 0 L/min. For all 45° BW tilt cases, the liquid film went beyond the second landmark of the OL region. This improved liquid flowability was presumably because of a steeper inclined wall than the 60° BW tilt cases. The flowability difference was particularly apparent in the liquid film morphology and translation distance after the first dose, with a tapered shape about 2 cm beyond the nasal valve for 45° BW tilt versus a rounded shape that barely went beyond the nasal valve (Figure 7a vs. Figure 6a). 

Significant differences were observed in the spray distribution between 30° and 45° BW tilt head positions (second panel vs. first panel in Figure 7a). Because of a steeper slope at a 30° tilt head position, the gravitational component after the first dose became stronger than the wall adhesion force, causing the liquid to move straight down along the side walls instead of adhering to the nasal roof like in all other cases considered earlier (second panel, Figure 7a). After applying the second dose, the steeper slope also led to lower retention in the front nose and a perceivable deposition in the back nose (second and third panels, Figure 7a). 

### 3.5. Effect of Inhalation Flow Rate

The effect of inhalation flow rate on spray deposition was tested by considering 10 and 20 L/min. At 10 L/min (Figure 7b), the liquid film after the first dose traveled a greater distance because of the increased flow shear, which disrupted and mobilized the otherwise stagnant liquid film when there was no flow. A notable difference in deposition pattern was observed following the second dose administered at 10 L/min, with the sprays effectively covering most of the middle upper nose, as opposed to the coverage of the nasal roof solely with no flow. A quantitative comparison of the regional DF following one dose and two doses administrated at 10 L/min is shown in the rightmost panel of Figure 7b. As expected, the front nose retention rate following one dose was much higher than that following two doses for both head positions (45° and 60° BW tilt) considered. As a result, lower DFs in the middle upper nose were obtained following one dose than two doses. Spray deposition in the middle lower nose was observed following both one dose and two doses administrated at 10 L/min with a head position of 45° BW tilt (Figure 7b, middle and right panels). Its steeper slope of the nasal roof wall and the flow shear diverted a fraction of sprays to the middle lower passage. This was in contrast to the 60 BW tilt head position at 10 L/min, where no or negligible sprays were deposited in the middle lower passage following one dose and two doses. Therefore, spray deposition was more sensitive to the head position with a non-zero flow rate. Likewise, the DF variability at 10 L/min was much higher than without a flow (Figure 7b vs. Figure 5c). 

Figure 7c shows the spray dosimetry at 20 L/min. The high flow through the middle meatus transported the majority of the sprays to the middle lower nose, and only a small fraction to the middle upper nose. There was even a small amount of spray droplets deposited in the nasopharynx (yellow arrow, Figure 7c). Furthermore, a much higher degree of variability in dosimetry was observed at 20 L/min compared to 0 and 10 L/min, as evident by the quantitative dosimetry comparison in Figure 7c (right panel). 

### 3.6. Effects of Solution Viscosity

**Figure 8:**
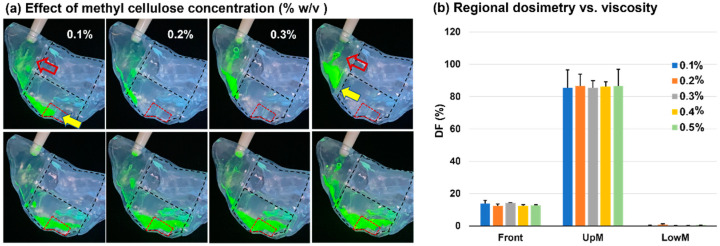
Effects of solution viscosity on the regional spray dosimetry with varying methyl cellulose concentrations (% *w*/*v*): (**a**) spray deposition distributions; (**b**) regional dosimetry vs. viscosity.

Figure 8 shows the effects of solution viscosity on spray dosimetry by varying the methyl cellulose (MC) concentration (0.1–0.5% *w*/*v*) in the solution. The correlation between the solution viscosity and the methyl cellulose concentration is shown in Figure 2. Following a single-dose application, the liquid film of a lower MC concentration traveled a greater distance along the nasal roof (solid yellow arrows, upper panel of Figure 8a). The remnant liquid films on the vestibular side walls appeared much thinner for 0.1% and 0.2% solutions than for the 0.5% solution (hollow red arrows, upper panel of Figure 8a). 

The spray dosimetries following the second dose of 0.1–0.5% *w*/*v* MC concentrations are visualized in the lower panel of Figure 8a and quantified in Figure 8b. Surprisingly, the two-dose DFs appeared to be insensitive to the solution MC concentration, with insignificant differences in both the front nose and middle-upper nose among the five solutions (Figure 8b). All liquid films reached or went beyond the posterior OL region, indicating a good coverage of the OL mucosa. Considering the 0.5% solution, its viscosity nearly doubled that of the 0.4% solution (16.1 vs. 31.8 mPa·s, Figure 2). However, the added mass from the second dose successfully mobilized the liquid dam accumulated in the front nose, which subsequently entered the superior meatus and ultimately reached the olfactory region. 

### 3.7. Left-Right Discrepancy in Dosimetry

**Figure 9:**
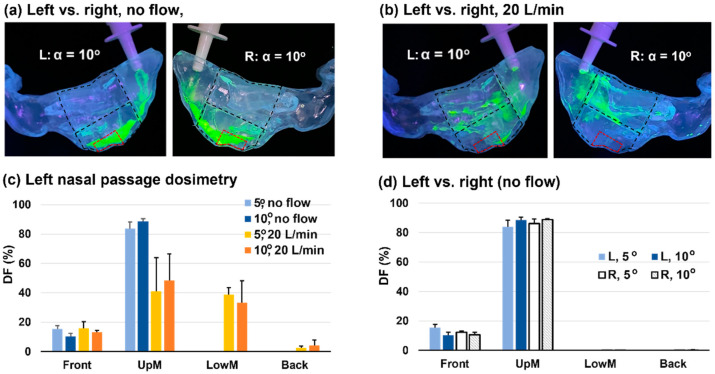
Comparison of nasal spray deposition between the left and right nasal passages: (**a**) surface deposition at the nozzle angle of 10° with no flow; (**b**) surface deposition at the nozzle angle of 10° at an inhalation flow rate of 20 L/min; (**c**) quantified regional dosimetry in the left passage at 0 and 20 L/min with nozzle angles of 5° and 10°; (**d**) comparison of dosimetry between the left and right nasal passages at 0 L/min.

A comparison of the spray dosimetry between the left and right nasal passages with a 60° BW tilt head position is shown in Figure 9 for a nozzle angle of 10° at two flow rates (0 and 20 L/min). The solution utilized had an MC concentration of 0.4% *w*/*v*. Overall, given all other variables being the same, the two passageways yielded comparable deposition distributions (Figure 9a,b) and regional DFs (Figure 9c,d). Note that the nasal cast model was developed from patient-specific MRI scans, and the two passages had different morphologies. At 0 L/min, the liquid film in the left passage, much like that in the right passage, extended over the olfactory region after two doses (Figure 9a). Distinct deposition patterns were observed at 20 L/min. The right passage had predominant coverage in the middle lower nose, while the left passage exhibited more coverage in the middle upper nose (Figure 9b). 

### 3.8. Image-Based Olfactory Dosimetry Estimation

**Figure 10:**
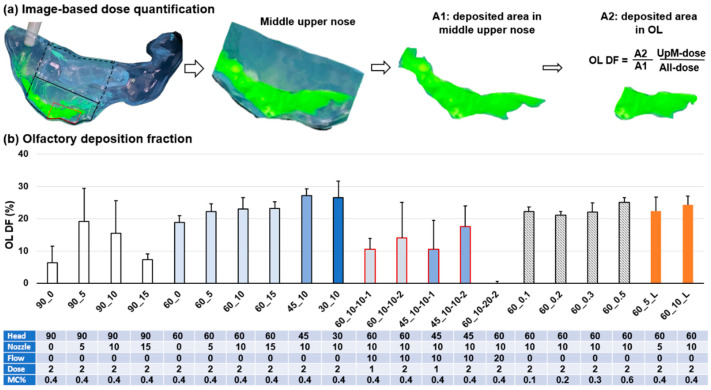
Image-based dosimetry: (**a**) estimation diagram and (**b**) olfactory deposition fraction.

The deposition fraction (DF) in the olfactory region was calculated as (A2/A1) times the DF in the middle upper nose, where A2 and A1 were the liquid-covered area in the OL region and middle-upper nose, respectively (Figure 10a). Figure 10b compares the OL DFs and standard deviations vs. the head position, nozzle angle, dose number, flow rate, MC concentration, and passage geometry. 

It was observed that the vertex-to-floor (90°) head position failed to yield the optimal OL DF and, at the same time, had a large variability in OL DF (the first four bars, Figure 10b). By contrast, the head position of 60° and 45° gave satisfactory OL DFs except for the nozzle angle of 0° (60°: four light blue bars and 45°: one blue bar in Figure 10b). Their standard deviations were also lower. Thus, a head position ranging 45–60° and a nozzle angle ranging 5–10° (with two-dose application and no inhalation flow) were proposed for effective delivery of nasal spray to the OL region. The head position of 30° also had the potential to dispense high doses to the OL region (deep blue bar, Figure 10b); however, it was not recommended because of its relatively high variability in OL DF and high DF in other regions, as previously explained in Figure 7a.

A lower magnitude and higher variability in the OL DF were observed for the inhalation flow rate of 10 L/min and 20 L/min, and for single-dose applications (red boundary bars). With two-dose applications, the OL DF and variability did not exhibit high sensitivity to the solution viscosity ranging 1.9–32 mPa·s, nor to the left or right nasal passage in this study (rightmost six bars, Figure 10b). After putting everything together, it was suggested that for optimal delivery of nasal spray to the OL region, a head position between 45° and 60° BW tilt from supine and a nozzle angle between 5° and 10° normal to the nostril angle should be used, along with a two-dose application and no inhalation flow.

### 3.9. Deposition Sensitivity Analysis to Delivery Variables

**Figure 11:**
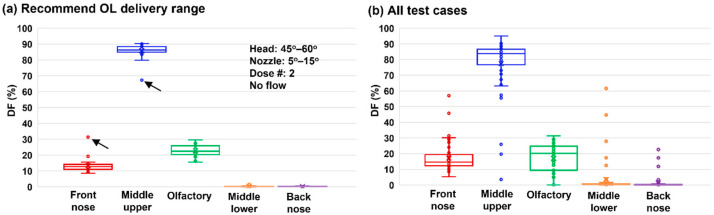
Box plots: (**a**) middle-upper nose coverage variability (overall, vs. head orientation, and vs. nozzle degree), and (**b**) comparison of deposition variability among five regions.

Figure 11a shows the box plots of the nasal spray dosimetry variability among five regions based on the recommended delivery variables. Based on the nasal model used in this study, a DF of 22.7 ± 3.7% was achieved in the strictly defined OL region that was bounded by two landmarks, as illustrated in Figure 1a. It is worth noting that in previous studies, the OL region had been defined to cover varying extents of the top nose, and the defined OL area/location could significantly affect the OL dose estimation. Further examination of Figure 11a revealed that the two worst outliers (black arrows) came from one single test case, which had a 60° tilt head position and a nozzle angle of 15°. This was consistent with the abnormally large DF variability in Figure 5c for the front nose and middle-upper nose.

The DF variability based on all test cases is shown in Figure 11b. As expected, a much higher variability and more outliers were observed for the DF in all five regions. Individual checks of these outliers showed that they mainly came from test cases with 20 L/min or single-dose administration, suggesting that these two categories should be excluded. 

**Figure 12:**
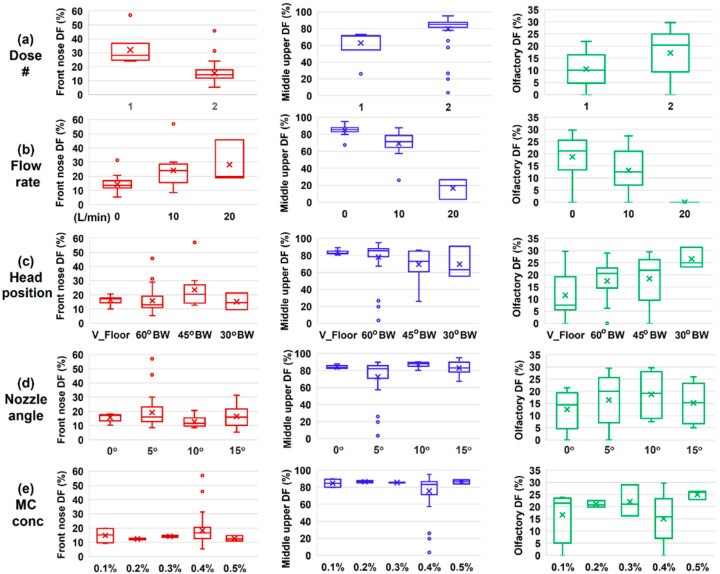
Box plots of the regional deposition fractions from sixty test cases in response to varying delivery variables: (**a**) the number of applied doses, (**b**) inhalation flow rate, (**c**) head orientation, (**d**) nozzle angle, and (**e**) spray solution viscosity by varying methyl cellulose concentrations. Regions of interest: front nose, middle-upper nose, and olfactory region.

Sensitivity analyses of the regional DF to individual delivery variables are presented in Figure 12a–e for the dose number, flow rate, head position, nozzle angle, and MC concentration, respectively. The front nose DF was noted to be highly sensitive to the number of doses applied and the inhalation flow rate, both of which exhibited large variations in the mean value and box size (first column, Figure 12). By comparison, the front nose DF was relatively insensitive to the nozzle angle (0–15°) and solution viscosity (1.9–32 mPa·s). Considering the middle-upper nose DF (second column, Figure 12), the highest variability occurred when the flow rate varied, while the lowest variability occurred with varying nozzle angle and solution viscosity.

The third column of Figure 12 shows the OL DF sensitivity analysis to input variables. Its y-coordinate range was 0–35% in contrast to 0–60% in the first column and 0–100% in the second column. It was clear that a two-dose application could significantly improve OL DF over a single-dose application (Figure 12a). Furthermore, the presence of an inhalation flow reduced the OL DF, and a flow rate of 20 L/min could completely preclude dispensing nasal sprays to the OL region (by diverting to the middle meatus), as shown in Figure 12b. No, or very slow, flow was expected to attain the optimal OL delivery, where the gravity-driven liquid film translocation along inclined walls was the major mechanism for delivering drugs to the OL region. In Figure 12c, the vertex-to-floor (V_Floor) head position was shown to deliver significantly lower doses to the OL region than the other three head positions and, thus, should not be used for future OL delivery. In Figure 12d, the nozzle angle of 5° and 10° overall performed better than 0° and 15° in OL delivery. Again, the OL DF in this study did not exhibit significant sensitivity to the viscosity of the solution (Figure 12e), given that two doses were applied, which triggered and facilitated the liquid film translocation. 

## 4. Discussion

One major difference between nasal sprays and nebulizers is the large size and high speed of the spray droplets, which, upon deposition in the front nose, will form a liquid film and cause dripping [51]. Using a transparent nasal cast and fluorescent imaging allows a detailed examination of the film’s dynamic behavior that had been previously veiled due to 3D printing materials. The real-time feedback on the input variables, in turn, enables prompt adjustments to individual delivery parameters. This interactive manner can notably expedite the design process for continuous improvement to reach an optimal delivery protocol. Similar delivery systems as in this study are promising as a test platform for other nasal spray applications such as para-sinus drug delivery and for studying the complex interactions among the patient-, device-, and administration-related variables. 

Different levels of sensitivity to delivery variables were observed for the dynamic liquid film translocation and final olfactory deposition. Applying two doses from a soft-mist inhaler (~125 mg per dose) was needed to destabilize the accumulated liquid film in the front nose and mobilize the fluid along the inclined nasal roof toward the olfactory region. However, applying more than two doses would over-flood the olfactory and cause waste in the nasopharynx. The inhalation flow was observed to decrease the liquid film translocation to the OL region, which diverted a portion of the fluid to the middle meatus. At 20 L/min, almost no fluid reached the OL region. Considering the head position, our results showed that vertex-to-floor was not the best position for OL delivery, giving lower OL deposition and higher variability than the head position of 45–60° tilted backward from the supine position. Considering the nozzle angle, a range of 5–10° gave rise to higher OL deposition and lower variability than 0° and 15°. As a result, the following parameters were proposed to effectively deliver nasal sprays to the OL region: a head position tilting 45–60° backward from the supine position, a nozzle angle ranging 5–10° counterclockwise from the nostril normal, two doses, and no inhalation flow. In practice, the patient should breathe slowly during the spray application and hold their head position for at least 60 s to allow the liquid film to translocate to the OL region.

The results of this study demonstrated that delivering clinically significant doses to the olfactory (OL) region is feasible by leveraging the liquid film translocation after nasal spray administration. With recommended delivery parameters, a delivery efficiency of 22.7 ± 3.7% was achieved in the strictly defined OL region. Note that the OL area hereof was around 20% of the middle upper nose and 8.7% of the middle nose. Different definitions of the olfactory region have been used in previous studies. While the general consensus is that the olfactory mucosa is situated at the top of the nose, there is no agreement on the precise location and extent where drug molecules can enter the brain. The absence of agreement makes it challenging to compare findings among various studies. Various areas have been reported for the olfactory region, which ranged from 5.0–6.8 cm^2^ [39,52], 10 cm^2^ [53], and 35 cm^2^ [47]. Similarly, the location and extent of the olfactory region have been defined differently. Si et al. [54] and Shi et al. [55] defined a crescent-shaped olfactory region at the apex of the nose. Schroeter et al. [47] defined an olfactory region with a similar area but different locations, which was slightly posterior to the nasal apex and was similar to the definition in this study. An OL delivery efficiency of 5% was reported using 10.3 μm particles. On the other hand, gamma scintigraphy studies commonly regarded the superior meatus as synonymous with the olfactory zone [56,57,58,59]. Using a nasal cast composed of three parts (lower, middle, and top) and a nasal pump, Wang et al. [32] measured a remarkable delivery efficiency of 73.5% to the top part, which had been described as the olfactory zone. This high percentage, however, was close to the spray deposition fraction of 80% in the middle upper nose in this study. More recently, Si et al. [12] numerically studied the liquid film translocation in the nose and predicted a 6.2% delivery efficiency to the olfactory region with the same definition as in this study. However, the liquid film translocation path in [12], which was linear from the vestibule to OL, was different from this study (along the nasal crest). A standard definition of the location and scope of the olfactory region is necessary for nose-to-brain drug delivery and especially for the performance evaluation of nasal devices targeting the olfactory region. 

In this study, we observed that two doses were sufficient to mobilize the liquid films of all methyl cellulose (MC) concentrations (0.1–0.5% *w*/*v*) considered with appropriate head and device orientations. Theoretically, the added mass to mobilize the liquid film formed from the first dose would vary for formulations of different MC concentrations (or viscosities). In this study, the formulation viscosity ranged 1.9–31.8 mPa·s (Figure 2), which covered most nasal spray formulations. It was thus expected that two doses would be sufficient for olfactory delivery for most nasal sprays in practice. We have not quantified the exact mass needed to mobilize the first-dose film because the soft-mist inhaler could only release a specific mass (~125 mg) per dose. To this aim, a device that can release a small mass is needed by progressively adding incremental masses till the liquid film starts to move. This aim, however, was out of the scope of this study.

The limitations of this study include a single airway, 3D-printed rigid nasal casts, and a limited number of test cases. Both the initial deposition and subsequent liquid film translocation were sensitive to the geometrical details of the nasal cavity. Thus, inter-subject variability is expected. In this study, the geometrical effects were evaluated by comparing the liquid film translocation and OL delivery between the right and left nasal passages of the nasal cast. It was observed that despite the large differences in the transient behaviors of the liquid film motion, the final dosimetry (and even variability) in the OL region were similar between the two passages. Future studies are needed in a sufficiently large cohort of nasal models that are representative of the targeted patient group, such as age, gender, race, and health condition. Not all factors were explicitly investigated, such as fluid density and surface tension, despite these two factors varying with MC concentrations. Another interesting question that needs further investigation is the liquid film stability on an inclined wall of different materials. The liquid film behavior on the nasal epithelium will differ from that of the 3D printing SLA material because of their differences in surface energy, roughness, and interactions with the fluid [60]. This question was partially answered in this study by considering the spray solutions of varying methyl cellulose (MC) concentrations (0.1–0.5% *w*/*v*). It was demonstrated that with a sufficiently steep slope (45–60° head position) and two-dose application (i.e., large enough weight to trigger the film motion), the MC concentration had an insignificant effect on the final OL dosimetry (Figure 8b). However, we also observed different shapes and motion speeds of the liquid film among different solutions after applying both the first and second doses. Future studies of the fluid and cast material effects on transient fluid translocation and OL deposition in different nasal airway models were needed. The soft-mist inhaler used in this study generated polydisperse droplets with a wide size distribution (Figure 3). Thus, deposition images with monodisperse aerosols were not available. We refer interested readers to a study by Perkins et al. [61], who computationally studied nasal deposition with different monodisperse aerosol sizes. 

## 5. Conclusions

In summary, an in vitro system for nasal spray delivery was developed using sectional, transparent nasal casts and fluorescent imaging. A systemic study of the delivery parameters was conducted for effective olfactory (OL) delivery, which included head position, nozzle angle, dose number, inhalation flow rate, and spray solution viscosity. The transient liquid film translocation, the final deposition in the olfactory region, the deposition variability, and the input sensitivity were examined. Specific findings are:

(1) The OL dosimetry depended not only on the initial deposition of spray droplets but also on the liquid film translocation.
(2) A two-dose application from the soft-mist inhaler was needed to mobilize the liquid film and enable it to move to the olfactory region.
(3) Recommended OL delivery parameters included: a head position tilting 45–60° backward from the supine position, a nozzle angle ranging 5–10° counterclockwise from the nostril normal, two doses, and no inhalation flow.
(4) With the recommended protocol, a delivery efficiency of 22.7 ± 3.7% was achieved in the strictly defined OL region.
(5) The presence of inhalation flow reduced the liquid film translocation to the OL region, with negligible OL doses at 20 L/min.
(6) The vertex-to-floor head position was not optimal for OL delivery, with lower OL delivery efficiency and higher variability.

## Data Availability

The data presented in this study are available on request from the corresponding author.
